# Differential Mucin Expression by Respiratory Syncytial Virus and Human Metapneumovirus Infection in Human Epithelial Cells

**DOI:** 10.1155/2015/347292

**Published:** 2015-04-22

**Authors:** Ma. Del Rocío Baños-Lara, Boyang Piao, Antonieta Guerrero-Plata

**Affiliations:** ^1^Department of Pathobiological Sciences, Louisiana State University, Baton Rouge, LA 70803, USA; ^2^Center for Experimental Infectious Disease Research, Louisiana State University, Baton Rouge, LA 70803, USA

## Abstract

Mucins (MUC) constitute an important component of the inflammatory and innate immune response. However, the expression of these molecules by respiratory viral infections is still largely unknown. Respiratory syncytial virus (RSV) and human metapneumovirus (hMPV) are two close-related paramyxoviruses that can cause severe low respiratory tract disease in infants and young children worldwide. Currently, there is not vaccine available for neither virus. In this work, we explored the differential expression of MUC by RSV and hMPV in human epithelial cells. Our data indicate that the MUC expression by RSV and hMPV differs significantly, as we observed a stronger induction of MUC8, MUC15, MUC20, MUC21, and MUC22 by RSV infection while the expression of MUC1, MUC2, and MUC5B was dominated by the infection with hMPV. These results may contribute to the different immune response induced by these two respiratory viruses.

## 1. Introduction

The mucosal surface of the respiratory tract is protected by a layer of mucus [1], a viscoelastic, gel-like substance that covers the epithelial surface of various mammalian tissues, including the respiratory tract. The main functions of mucus include protecting the epithelial surface from injury, by facilitating removal of materials that enter the lung and in the pathogenesis of many lung diseases, particularly those involving chronic inflammation of the airways or susceptibility to infection. The viscous and elastic properties of mucus gel are generally attributable to the physical properties and structural features of mucin (MUC). MUC are high molecular mass, highly glycosylated macromolecules that are the major components of mucus secretions [2]. To date, 22 MUC proteins have been described in human; according to their subcellular localization they are classified in two groups: membrane-bound MUC, which have a transmembrane domain that anchors them to the cell membrane. The members in this group are MUC1, MUC3A/B, MUC4, MUC11, MUC12, MUC13, MUC15, MUC16, MUC17, MUC18, MUC20, MUC21, and MUC22 and secreted mucins: MUC2, MUC5AC, MUC5B, MUC6, MUC7, MUC8, and MUC19. MUC9 is both located in the cell surface and secreted as well. Except for MUC6, MUC7, and MUC17, all the mucins abovementioned are expressed in the airways [3–5]. High production of mucus is a characteristic in inflammatory lung diseases such as bronchial asthma, chronic obstructive pulmonary disease, bronchiectasis, and cystic fibrosis [5]. Moreover, overexpression of MUC has been reported in several malignancies like breast, gastric, colorectal, pancreatic, lung, small bowel, and ovarian cancers [6, 7].

Respiratory syncytial virus (RSV) is a negative sense single stranded RNA virus member of the Paramyxoviridae family and* Pneumovirus* genus that is the main cause of severe lung disease in infants and young children. It is predicted that, by the age of 2, all the children will have experienced an infection with RSV [8]. Human metapneumovirus (hMPV) is another member of the same family under the* Metapneumovirus *genus and is also a considerable cause of respiratory illness in children, immunocompromised patients, and elderly. It accounts up to 10% of the hospitalizations due to respiratory viral infections. By 5 years of age, virtually all the children have been infected [9]. RSV and hMPV have a seasonal distribution, being usually isolated during the winter time and are associated with both upper and lower respiratory tract infections in children and adults. Despite these viruses having a strong impact in public health, there is not commercial vaccine available. The clinical characteristics observed in patients infected with hMPV or RSV are practically indistinguishable ranging from mild common cold-like symptoms to more severe lower respiratory tract illness including pneumonia and bronchiolitis [10–12]. However the immune response elicited by these two viruses in infected individuals can be significantly different, as indicated by several studies analyzing the cytokine production in nasal washes from infant with primary infection of RSV or hMPV. Reported data have shown that the concentration of inflammatory cytokines is higher in patients infected with RSV than in those with hMPV [13]. Moreover, RSV and hMPV induce a distinct T helper cytokine profile in infected children [14, 15], suggesting that, despite the structural and pathogenic similarities between RSV and hMPV, the immune response elicited by these respiratory human paramyxoviruses is virus specific. Therefore, fundamental aspects of the immune response to RSV and hMPV infections need further research.

In this work, we compare the MUC expression between RSV and hMPV infection in human epithelial cells. Our data demonstrate a significant differential MUC expression by RSV and hMPV, confirming that these two viruses induce a different immune response.

## 2. Materials and Methods

### 2.1. RSV and hMPV Preparations

hMPV (strain CAN97-83) stock was provided by the Respiratory Virus Section, Centers for Disease Control (CDC), Atlanta, GA, with permission from Dr. Guy Boivin at the Research Center in Infectious Diseases, Regional Virology Laboratory, Laval University, Quebec City, Canada. Virus was propagated and titrated in LLC-MK2 cells (ATCC CCL7) in the presence of trypsin (Worthington, Lakewood, NJ), as described elsewhere [16]. RSV A2 was grown in HEp-2 cells (ATCC CCL-23). The virus titer was determined by methylcellulose plaque assay [17, 18]. Both viruses were purified by polyethylene glycol, followed by centrifugation on discontinuous sucrose gradients.

### 2.2. Infection of Epithelial Cells

A549 cells (ATCC CCL-185) were cultivated in F12K medium enriched with 10% of FBS and with 1% of penicillin-streptomycin. Cells were infected with hMPV and RSV at a multiplicity of infection (MOI) of 3. At the indicated time points, cells were collected and processed for RNA isolation.

### 2.3. Real-Time Quantitative Reverse Transcription-PCR (qRT-PCR)

RNA from cells was extracted using the RNAeasy-plus kit (Qiagen). Determination of the expression of the genes by qRT-PCR was performed by using predesigned TaqMan assays, as previously described [19]. All primers and probes were obtained from Integrated DNA Technologies. qRT-PCRs were run on the 7900HT fast real-time PCR system following the manufacturer's suggested cycling parameters (Applied Biosystems). The comparative cycle threshold (ΔΔCT) was used to quantitate the expression of target genes and was normalized to the endogenous reference (GAPDH) expression levels of transcripts from uninfected (control) cells.

### 2.4. Statistical Analyses

Statistical analyses were performed with the GraphPad Prism 5 software, using a one-way Analysis of Variance (ANOVA) followed either by Tukey post test to ascertain differences between hMPV and RSV groups, or by Dunet post test to analyze differences between hMPV and RSV infected cells against uninfected cells. Results are expressed as means ± SEM.

## 3. Results and Discussion

Mucus is a critical part of the physiological and pathological processes in the airways. It can protect the respiratory tract as a physical barrier to remove inhaled insults. However, excessive production of mucus in the lung can contribute to inflammatory processes. Although mucins (MUC) are the main component of mucus, their response in respiratory viral infections is still largely unknown. Therefore, in this work we investigated the MUC response to RSV and hMPV, two closest related human paramyxoviruses, which despite their shared structural and epidemiological characteristics differ in many aspects of the immune response they trigger in the infected host. Knowledge of the host immune response induced by RSV and hMPV is critical to understanding the viral pathogenesis of these highly relevant respiratory viruses.

**Figure 1:**
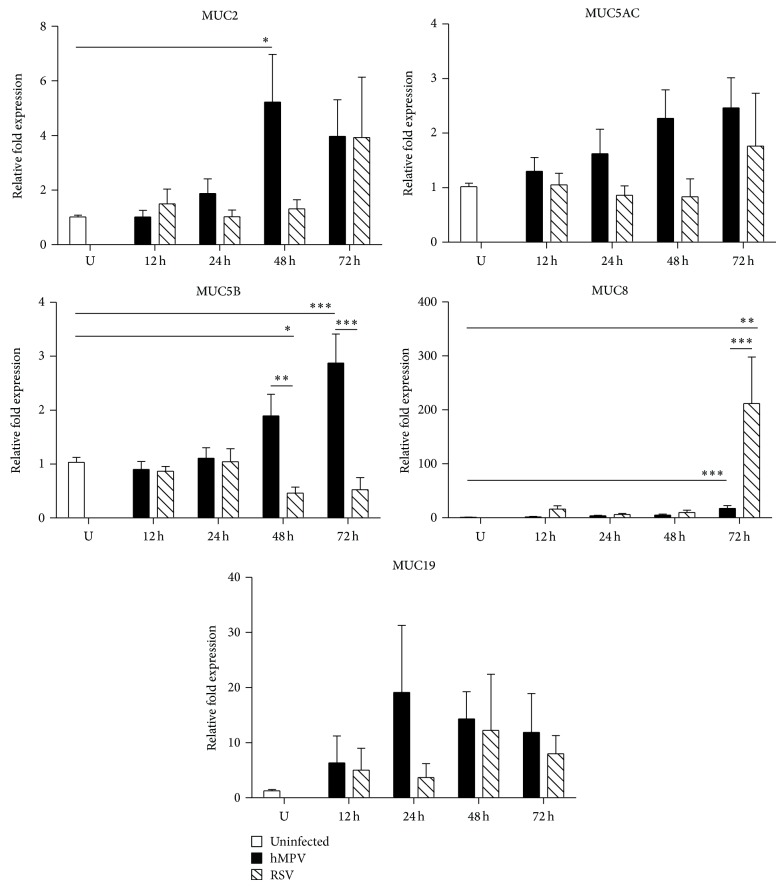
Secreted mucins expression in A549 by hMPV and RSV infection. Cells were infected with hMPV or RSV at an MOI of 3 for 12, 24, 48, or 72 h. RNA was isolated, and the expression of MUC genes was determined by qRT-PCR. Data shown in the graphs are the mean of 5 independent experiments. Statistical significant differences are indicated. ^∗^ *P* < 0.05; ^∗∗^ *P* < 0.01; ^∗∗∗^ *P* < 0.001.

In order to evaluate the profile of mucins expression in hMPV and RSV infection, human alveolar epithelial cells (A549) were infected with RSV or hMPV at a multiplicity of infection (MOI) of 3. RNA was collected at 12, 24, 48, and 72 h after infection and the expression of the following mucins MUC1, MUC2, MUC3, MUC4, MUC5AC, MUC5B, MUC8, MUC13, MUC15, MUC16, MUC19, MUC20, MUC21, and MUC22 was analyzed by qRT-PCR. Our results demonstrated that hMPV induced a significantly different mucin expression than that of RSV. As shown in Figure 1, the expression of secreted mucins was induced by hMPV infection as indicated by significant upregulation of MUC2 (48 h 5.22 ± 1.75-fold), MUC5B (48 h 1.89 ± 0.40- and 72 h 2.87 ± 0.53-fold), and MUC8 (48 h 5.19 ± 1.68- and 72 h 17.44 ± 5.19-fold) when compared to uninfected control cells, while RSV only induced significant upregulation of MUC8 gene (72 h 212.0 ± 86.14-fold) and marginal expression of MUC2, MUC19, and MUC5AC. In line with our data, the expression of MUC5AC has also been observed in RSV-infected human bronchial epithelial cells [20]. MUC5AC is a major gel-forming mucin expressed in the lung that has been reported to confer a protective effect during viral infections as demonstrated in influenza virus infection using a Muc5ac-transgenic mouse model [21]. MUC8 was originally identified in submucosal glands in human tracheal epithelium [22] but, more recently, its expression has been reported in epithelial cells stimulated with LPS [23] and rhinovirus infection [24, 25]. The expression of MUC8 appears to be dependent on activator protein-2 alpha (AP2*α*) [26], prostaglandin E2 [27], and IL-1*β* [28], suggesting that this mucin is regulated by the inflammatory immune response. However, whether MUC8 participates in the inflammatory processes during hMPV and RSV infections warrants further research. Overall, our data indicate that hMPV induces a stronger response of MUC2, MUC5AC, and MUC5B while RSV induced ~200-fold increase of MUC8 when compared to that induced by hMPV infection.

**Figure 2:**
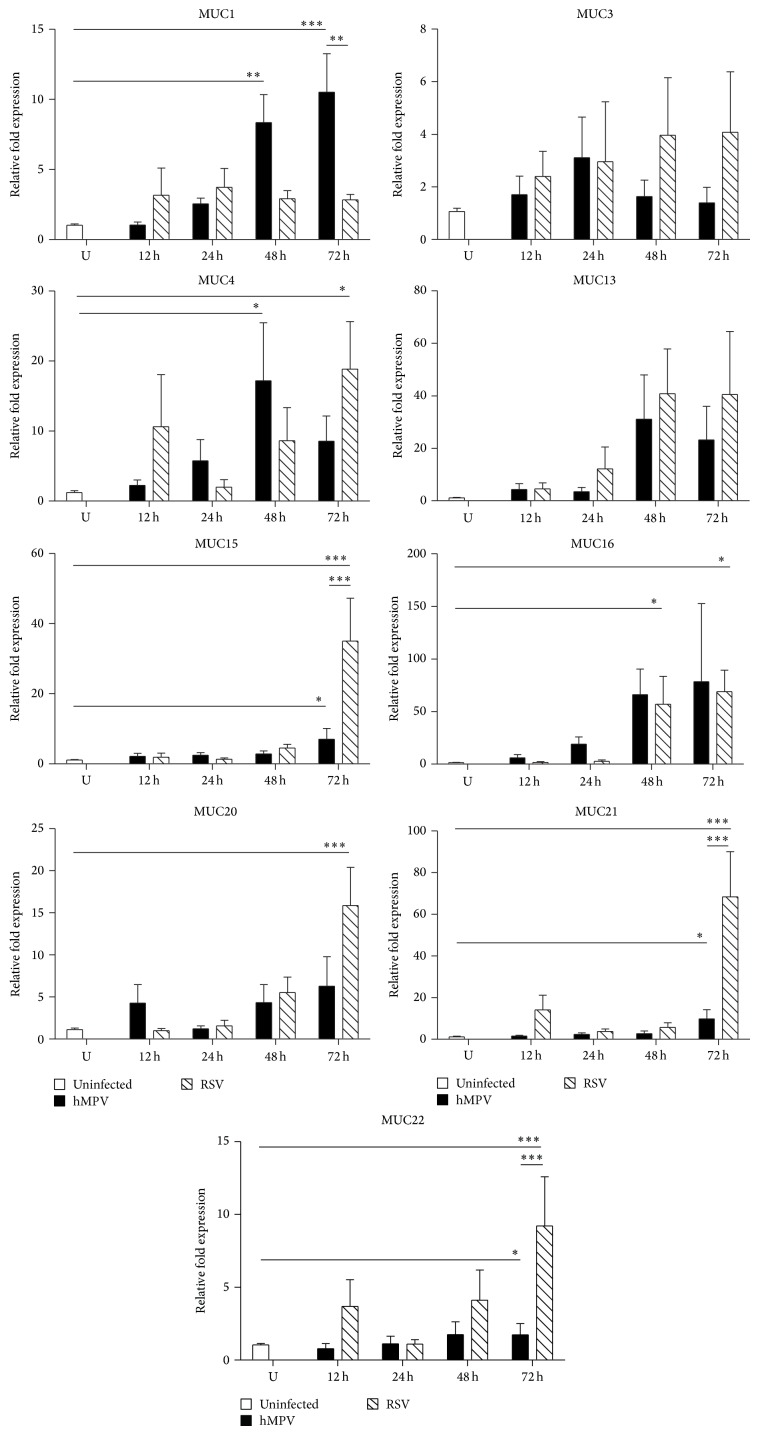
Cell-adhered mucins expression in A549 by hMPV and RSV infection. Cells were infected with hMPV or RSV at an MOI of 3 for 12, 24, 48, or 72 h. RNA was isolated, and the expression of MUC genes was determined by qRT-PCR. Data shown in the graphs are the mean of 5 independent experiments. Statistical significant differences are indicated. ^∗^ *P* < 0.05; ^∗∗^ *P* < 0.01; ^∗∗∗^ *P* < 0.001.

The expression of membrane-bound MUC was also explored in this work. As shown in Figure 2, the expression of MUC1 was significantly upregulated by hMPV (48 h 8.35 ± 1.99; 72 h 10.52 ± 2.73-fold) when compared to uninfected control cells (1.03 ± 0.09-fold) but only marginally induced by RSV (48 h 2.92 ± 0.57-fold; 72 h 2.82 ± 0.39-fold). Similar data have been reported by Li et al. who also found that MUC1 is induced by RSV in A549 cells at an MOI of 1 and 5 [29]; however, the levels of expression were higher than those observed in this work. On the other hand, RSV induced significant higher levels of expression of several MUC than those induced in epithelial cells infected with hMPV: MUC15 at 72 h (35.0 ± 12.2 versus 7.0 ± 3.1-fold), MUC20 72 h (15.87 ± 4.53 versus 6.28 ± 3.5-fold), MUC21 at 72 h (68.4 ± 21.6 versus 9.8 ± 4.3-fold), and MUC22 at 72 h (9.22 ± 3.37 versus 1.733 ± 0.7-fold). The expression of MUC4 and MUC16 was induced comparably by both viruses, while no significant expression of MUC3 and MUC13 was observed after RSV or hMPV infection. Together, these data indicate that hMPV is a stronger inducer of MUC1 than RSV, while RSV is a stronger inducer of MUC15, MUC20, MUC21, and MUC22 when compared with hMPV infection.

The differential MUC expression in response to RSV and hMPV, demonstrated in the present work, is in accordance with the differential response of several aspects of the immune response previously reported. We have shown that RSV and hMPV induce a distinct profile of cytokines and cellular activation in human dendritic cells [30]. Similar effect has been observed in the experimental mouse model where it has been suggested that hMPV induces a stronger immune response than that of RSV based on the higher percentage of neutrophils and NK cells recruited to the lung of the infected mice [31]. In fact, hMPV has also been found to be a stronger inducer of type I and type III IFN than RSV [16, 32, 33]. Moreover, hMPV induces stronger response of GM-CSF and KC than RSV. However, RSV-infected mice show higher levels of proinflammatory cytokines including IL-1, IL-6, and TNF-*α* than hMPV-infected mice [33]. Recent studies suggest that also the macrophage response plays a distinct role in RSV and hMPV infection as macrophages have been found to contribute to the pathogenesis of hMPV while they play a protective role in RSV infection [34]. Interestingly, immune differences between both infections are also influenced by age, as indicated by Oliver Schildgen and his group where young mice (4–6-week-old) showed a higher expression of NF-*κ*B and TNF-*α* expression when infected with hMPV and compared to those infected with RSV. However, the opposite effect was observed in older mice (19-month-old) [35]. Overall, these experimental evidences suggest that several aspects of the immune response elicited by these two viruses differ significantly.

## 4. Conclusions

These results demonstrate that RSV and hMPV induce a differential MUC expression, suggesting that this effect may contribute to the distinct immune response induced by these two respiratory viruses. However, very limited information related to the role of MUC exists, particularly on their role in respiratory viral infections. Therefore, further analysis related to the mechanisms of MUC expression in RSV and hMPV infection and their contribution to the immune response induced by these two highly relevant respiratory pathogens is warranted.
